# Radiological Diagnosis and Management of Epistaxis

**DOI:** 10.1007/s00270-013-0776-y

**Published:** 2013-11-15

**Authors:** Antonín Krajina, Viktor Chrobok

**Affiliations:** 1 Department of Radiology, University Hospital Hradec Kralove and Medical Faculty of Charles University, 500 05 Hradec Kralove, Czech Republic; 2 Department of Otorhinolaryngology and Head and Neck Surgery, University Hospital Hradec Kralove and Medical Faculty of Charles University, 500 05 Hradec Kralove, Czech Republic

**Keywords:** Posterior epistaxis, Selective percutaneous embolisation, Endovascular intervention

## Abstract

The majority of episodes of spontaneous posterior epistaxis treated with embolisation are idiopathic in nature. The angiographic findings are typically normal. Specific angiographic signs are rare and may include the following: a tumour blush, telangiectasia, aneurysm, and/or extravasation. Selective internal carotid artery (ICA) angiography may show rare causes of epistaxis, such as traumatic or mycotic aneurysms, which require different treatment approaches. Complete bilateral selective external and internal carotid angiograms are essential to evaluation. The images should be analysed for detection of central retinal blush in the external carotid artery (ECA) and anastomoses between the branches of the ECA and ICA. Monocular blindness and stroke are two of the most severe complications. Embolisation aims to decrease flow to the bleeding nasal mucosa while avoiding necrosis of the nasal skin and palate mucosa. Embolisation is routinely performed with a microcatheter positioned in the internal maxillary artery distal to the origin of the meningeal arteries. A guiding catheter should be placed in the proximal portion of the ECA to avoid vasospasm. Embolisation with microparticles is halted when the peripheral branches of the sphenopalatine artery are occluded. The use of coils is not recommended because recurrent epistaxis may occur due to proximal embolization; moreover, the option of repeat distal embolisation is lost. The success rate of embolisation therapy (accounting for late recurrence of bleeding) varies between 71 and 94 %. Results from endoscopic surgery are quite comparable. When epistaxis is refractory to nasal packing or endoscopic surgery, embolisation is the treatment of choice in some centres.

## Introduction

Haemorrhage from the nose is formally referred to as “epistaxis.” Haemostasis within the nose can be compromised by mucosal abnormalities, vessel pathology, or coagulation disorders. The aetiology of epistaxis is divided into groups: local and systemic factors. Local factors include the following: trauma, local inflammatory reactions, foreign bodies, postsurgical anatomical deformities, intranasal tumours, chemical inhalants, nasal-prong oxygen administration, and continuous positive airway pressure therapy for obstructive sleep apnoea. Systemic causes of epistaxis include the following: vascular disorders, especially hereditary haemorrhagic telangiectasia (HHT); blood dyscrasias; hematologic malignancies; and drugs affecting the normal clotting mechanism. Epistaxis arising from the anterior septal area (anterior epistaxis) is more common in children and young adults; it is the most common type of epistaxis and is more often venous in origin. This condition is usually responsive to treatment because it is readily accessible for nasal packing if bleeding does not resolve spontaneously [1, 2]. Therefore, endovascular therapy is usually not indicated for anterior epistaxis.

Approximately 5 % of epistaxis episodes arise from the posterior and superior portions of the nasal cavity, and the majority of posterior epistaxis episodes arise from the arteries of the septum. Posterior epistaxis is more common in older patients than in children [3] The most common factors associated with posterior epistaxis are hypertension [4, 5], acetylsalicylic acid or nonsteroidal anti-inflammatory drug use [6], previous episodes of epistaxis, alcohol use, and anticoagulant use. However, the majority of these cases are idiopathic [7]. Refractory epistaxis is defined as recurrent or persistent bleeding after appropriate packing or multiple episodes of epistaxis during a short period of time, each requiring medical attention. Embolisation for intractable epistaxis was first reported as an alternative to surgery and other methods by Sokoloff [8].

## Nonendovascular Therapy for Epistaxis

The management of epistaxis should begin with general measures, including calming the patient (with sedatives if necessary), application of a cold compress to the nape of the neck, decreasing the blood pressure if the patient is hypertensive, fluid resuscitation, and correction of any underlying coagulopathy. Local measures include the following: various methods of bleeding control, such as local application of haemostatic agents, infiltration of the bleeding area with vasoconstrictive agents, and cautery. Anterior nasal packing is performed by positioning strips of ointment-saturated gauze so as to produce satisfactory pressure on the bleeding mucosa. Posterior nasal packing is an option if anterior packing is insufficient to stop the haemorrhage or if the bleeding originates posteriorly. Posterior nasal packing requires general anaesthesia. A balloon catheter is inflated in the nasopharynx to stop the flow of blood posteriorly, and the anterior part of the nasal cavity is packed. The catheter coming out of the nose is fixed in place [3, 9].The patient should be monitored because posterior nasal packing can lead to nasal trauma, vasovagal reaction, and infection. Traditional gauze packing or balloon devices may be left in the nasal cavity for ≤48 h to prevent such complications. The success rate of posterior packing is reported to be between 48 and 83 %. Subsequent therapy can include either surgical endonasal coagulation or ligation [10]. An endoscopic approach has been used for direct electrocauterisation of the active bleeding site and endoscopic ligation of the sphenopalatine and ethmoidal arteries [11]. Surgical intervention is clearly indicated in anterior ethmoid bleeding because the ethmoidal arteries arise from the ophthalmic artery, the embolisation of which is generally considered to be dangerous.

## Endovascular Therapy of Epistaxis

### Relevant Anatomy

Detailed knowledge of the arterial anatomy of this region is important for safe and successful treatment. Stroke and blindness are two of the most severe complications. When retinal opacification from branches of the external carotid artery (ECA) is identified, embolisation must not be performed, and the patient should be referred for surgical treatment [12–15].

**Fig. 1:**
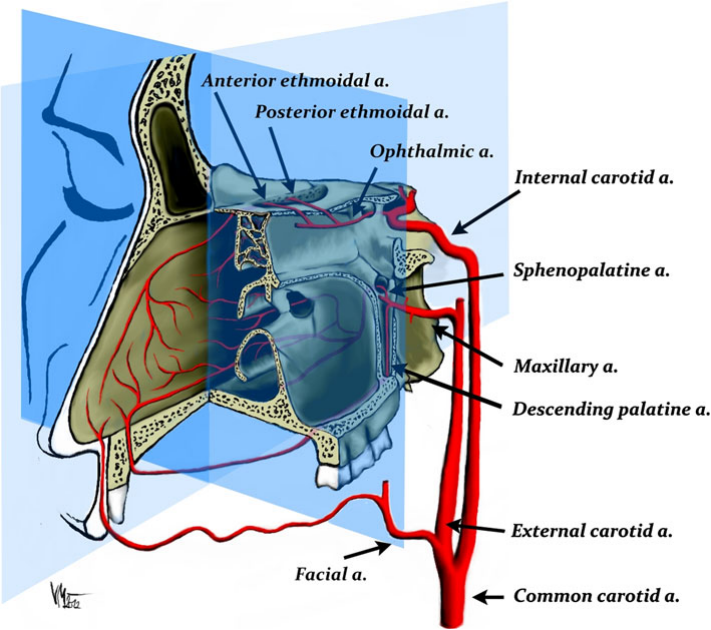
Schematic arterial supply of the sinonasal cavity. The majority of the posterior epistaxis episodes arise from the septum. The arterial branches involved in epistaxis include the internal maxillary artery, the facial artery, and the ophthalmic artery (Courtesy of V. Machova)

The arterial branches involved in epistaxis include the following: the internal maxillary artery (IMA), the facial artery, and the ophthalmic artery (Fig. 1). The other branches of the ECA are rarely involved.

**Fig. 2:**
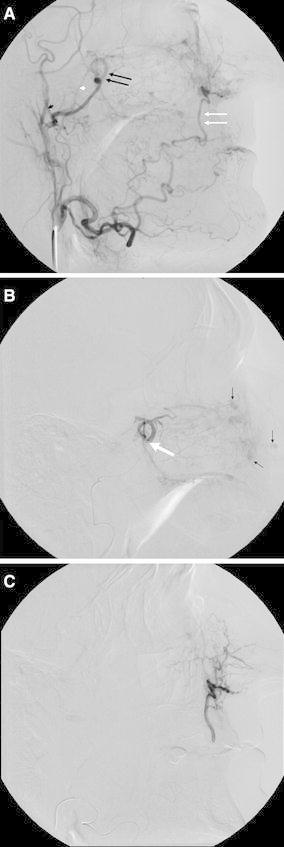
**A** External carotid angiogram of a patient with HHT and multiple episodes of severe epistaxis. The target artery for embolisation is the sphenopalatine artery (*double black arrows*) and the terminal portion of the facial artery (*double white arrows*). A microcatheter for embolisation should be placed distal to the middle meningeal artery (*small black arrowhead*) and accessory meningeal artery (*small white arrowhead*). **B** Selective internal maxillary angiogram showing the position of a microcatheter (*large white arrow*). There are separate mucosal hypervascular areas caused by telangiectasias (*small black arrows*). **C** Selective facial angiogram that shows the supply to the nasal cavity

The IMA is divided into three parts based on its relationship to the external pterygoid muscle [16, 17] (Table 1). The proximal portion is referred to as the “mandibular portion” and originates behind the neck of the mandible. The second, or pterygoid, portion is in contact with the external pterygoid muscle and varies in its location in relationship to this muscle. It is most commonly located superficial to the external pterygoid muscle, although in one third of cases, it is deep to this muscle. The middle meningeal artery arises in front of the inferior alveolar artery in the superficial variant and behind the inferior alveolar artery in the deep variant. The third, or pterygopalatine, portion is the terminal portion of the IMA, creating a loop within the pterygopalatine fossa. This vessel is the target for endovascular therapy in the treatment of posterior epistaxis (Table 2) (Fig. 2).

**Table 1:** Branches of the maxillary artery (modified according Allen et al. [16])

I. First (mandibular) portion
a. Anterior tympanic
b. Deep auricular
c. Middle meningeal^a^
d. Accessory meningeal^a^
e. Inferior alveolar
II. Second (pterygoid) portion
a. Deep temporal
b. Pterygoid
c. Masseteric
d. Buccal
III. Third (pterygopalatine) portion
a. Posterior superior alveolar
b. Infraorbital
c. Greater (descending) palatine^b^
d. Artery to foramen rotundum^a^
e. Artery of pterygoid canal (vidian artery)^a^
f. Pharyngeal
g. Sphenopalatine^b^

^a^Dangerous arteries for embolisation. The pterygoid artery and Vidian artery are considered to be two different branches by Lasjaunias et al. [20]

^b^These arteries are targets for embolisation

**Table 2:** Distribution of the third (pterygopalatine) portion of the maxillary artery (modified according Allen et al. [16])

Direction	Branch	Route of exit
Lateral	Posterior superior alveolar	Pterygomaxillary fissure
Anterior	Infraorbital	Inferior orbital fissure
Inferior	Greater palatine	Pterygopalatine canal
Medial	Sphenopalatine	Sphenopalatine foramen
Posterior	Artery of foramen rotundum	Foramen rotundum
	Artery of pterygoid canal	Pterygoid canal
	Pharyngeal	Palatinovaginal canal

**Fig. 3:**
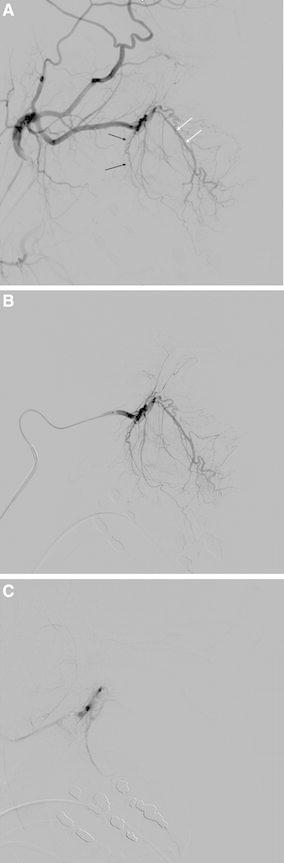
**A** Angiographic anatomy of the distal internal maxillary artery. The descending palatine artery (*black arrows*) outlines the posterior wall and floor of the maxillary antrum. The infraorbital artery (*double white arrows*) enters the orbit through the infraorbital fissure. **B** Selective distal internal maxillary artery angiogram in a lateral view. **C** Completion angiogram after embolisation with microparticles (Courtesy of J. J. Vitek)

The sphenopalatine artery, which is the branch of the third part of the IMA, leaves the pterygopalatine fossa by way of the sphenopalatine foramen [18] (Fig. 3). This artery is the major arterial supply to the nasal fossa and enters this fossa posteriorly to the superior meatus. Its lateral nasal branches supply the nasal turbinates, and the medial branches supply the nasal septum [19].

The descending palatine artery, another branch of the pterygopalatine portion of the IMA, supplies the nasal fossa. This artery courses through the pterygopalatine canal and emerges from the palatine foramen. It outlines the posterior wall and floor of the maxillary antrum. The terminal branch enters the incisive foramen and supplies the inferior nasal septum. Here, the branch anastomoses with the medial nasal branches of the sphenopalatine artery. The infraorbital artery enters the orbit through the infraorbital fissure and runs through the infraorbital foramen. Its branches supply the cheek, lower eyelid, and upper lip, and nose. This artery does not supply the nasal mucosa.

The ophthalmic artery gives rise to a collateral network with the sphenopalatine artery by way of the anterior and posterior ethmoidal branches. These arteries pass through the cribriform plate of the ethmoid. The superior labial artery, a small branch of the facial artery, contributes to the arterial supply of the nasal septum. This branch is not regularly visible on angiographic studies.

**Fig. 4:**
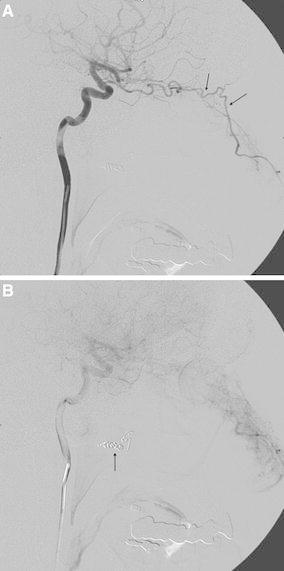
**A** Internal carotid angiogram in a patient with recurrent epistaxis. There is a rich collateral supply to the nasal cavity from the ophthalmic artery (*arrows*). **B** The cause of such a collateral pathway is previous proximal embolisation of the internal maxillary artery using coils (*arrow*) (Courtesy of Dr. M. Vavrova)

The facial artery is in hemodynamic balance with the other branches of the ECA. The preferred arterial supply is established in accordance with local hemodynamic needs [20]. This situation means that the direction of blood flow through anastomoses among the facial, internal maxillary, and ophthalmic arteries depends on the force and rate of injection, proximal vasospasm, and pre-existing occlusion due to ligation or embolisation [15] (Fig. 4).

Some investigators state that a venous plexus, called Woodruff’s plexus, is a common source of bleeding in cases of posterior epistaxis. This plexus is located in the posterior part of the inferior meatus and around the choanae [21].

### Endovascular Therapy for Idiopathic Posterior Epistaxis

#### Preprocedure Preparation

Computed tomography (CT) or magnetic resonance imaging is not typically indicated for the investigation of epistaxis unless tumour or other local diseases are suspected as an underlying cause. Idiopathic epistaxis is classified after exclusion of local or systemic causes. The interventional radiologist should be aware of the laterality of the nose bleed, the current severity of bleeding, and the patient’s history. These factors are crucial in deciding whether general anaesthesia is required for the procedure. General anaesthesia is routinely used only in children, noncompliant patients, and patients having uncontrolled epistaxis with possible compromise of the airway. The majority of embolisation procedures can be performed with intravenous sedation and analgesia with standard monitoring of blood pressure, electrocardiogram, and pulse oxymetry. Nasal packing, which is radiopaque, should be avoided in patients who might be candidates for embolotherapy.

#### Underlying Coagulopathy

The presence of any underlying coagulopathy should be evaluated because embolisation with polyvinylalcohol (PVA) microparticles is more effective in patients with normal platelet and plasma protein functions [22].

A proposed protocol for anticoagulants and epistaxis is as follows [1, 23]:

1.: Obtain full blood count, platelet count, and international normalised ratio (INR)
2.: Patients with metallic heart valves on warfarin should be kept only on a therapeutic level of INR. A cardiologist should be contacted if life-threatening bleeding occurs
3.: Patients with coronary stents treated with acetylsalicylic acid or other antiplatelet therapy should be discussed with a cardiologist for possible platelet transfusion in cases of life-threatening epistaxis

#### Angiography and Embolisation

Complete selective external and internal carotid angiograms are essential to evaluation. Angiograms should be analysed for the detection for arterial variants and anastomoses between branches originating from the external carotid and internal carotid arteries [14, 15]. Standard catheterisation of the common carotid artery (CCA) is performed. Depending on the type of the aortic arch, various diagnostic and guiding catheters are selected. The most frequently used are vertebral, Berenstein, Davis, Simmons, or Vitek catheters. A 5F or 6F guide catheter permits angiography while a microcatheter is in the target artery. A standard continual pressure flush with heparinised normal saline must be used to prevent the backflow of blood into the guiding catheter.

The first injection of 7–10 ml of iodinated contrast medium into the CCA is performed using the posterioanterior and lateral views. The angiogram should show any potential arterial pathology at the common carotid artery bifurcation, including atherosclerotic plaque or occlusion of the internal carotid artery (ICA) (Table 3). Because the majority of posterior epistaxis episodes treated by embolisation are idiopathic, angiographic findings in these cases may be normal. Specific angiographic signs are rare and may include the following: tumour blush, telangiectasia, traumatic pseudoaneurysm, and even contrast extravasation. Selective ICA angiography may show other sources of epistaxis, such as mycotic or traumatic aneurysm [12, 13].

**Table 3:** Angiographic and embolisation protocol in epistaxis

A. The ICA angiogram
1. Start on the side of epistaxis (if identified)
2. Check the carotid bifurcation for stenosis or occlusion
3. Look for central retinae blush, ophthalmic retina feeders to the nasal mucosa
4. Exclude source of epistaxis from the ICA
B. The ECA angiogram
1. Position the guide catheter with its tip just within the ECA to avoid vasospasm
2. Exclude filling of the ophthalmic artery (retinae blush on lateral view)
3. Exclude collaterals to the ICA
4. Show any arterial pathology as a source of bleeding
C. Embolisation of the IMA
1. Place microcatheter into the pterygopalatine portion of the IMA sufficiently distal to the accessory or middle meningeal arteries, preferably distal to the infraorbital artery
2. Perform flow-directed embolisation with PVA particles (250–500 μm) in nondiluted contrast agent using a 1-ml syringe
3. Avoid reflux and coil embolisation
D. Perform contralateral ICA and ECA angiograms as well as IMA embolisation
E. Remove the nasal packing and check the nasal pathway for bleeding; if no bleeding appears for 15 min, remove the microcatheter and perform a completion ECA angiogram
F. Perform embolisation of the ipsilateral facial artery with the microcatheter placed as distally as possible (at least distal to the submandibular part of the facial artery) using 250- to 350-μm PVA particles if significant contribution to the nasal mucosa is angiographically visible

Arterial vasospasm is an undesired but common consequence of forceful manipulation, particularly manipulation with the tip of the guiding catheter in the proximal part of the ECA or microwire manipulation in the branches of the ECA. The presence of proximal vasospasm impairing the arterial flow in the access artery can alter the hemodynamic pattern of the arterial feeders in the target area, thus rendering embolisation impossible. Vasodilators may be administered intra-arterially to treat or prevent vasospasm [24, 25].

**Fig. 5:**
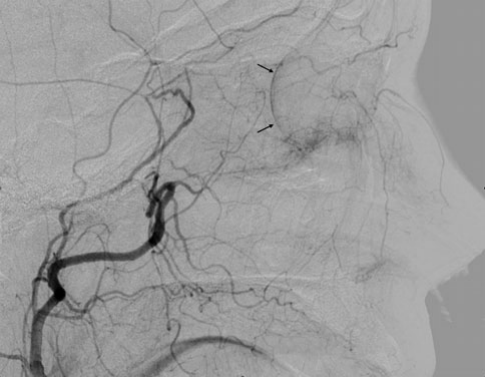
External carotid angiogram showing the supply of the retina (*arrows*)

A microcatheter that will allow the use of microparticles ≤500 μm in diameter is placed coaxially in the pterygopalatine segment of the IMA, and angiography is performed to determine if there are any large anastomoses involving the ICA or ophthalmic artery. The most useful projection to evaluate the ECA, IMA, and retinal blush is the lateral view (Figs. 4B, 5). Most commonly, supply of the ophthalmic artery from the ECA branches occurs due to collateralisation observed in chronic ICA stenosis or occlusion. The terminal branches of the pterygopalatine segment are best seen in frontal views.

Microparticle embolisation is performed using a standard free-flow technique. A wedged position of the microcatheter should be avoided (Fig. 3B). Ideally, the capillary bed of the nasal cavity is slowly saturated with embolic microparticles. This goal is accomplished using fluoroscopic guidance in the lateral view to assess blood flow velocity in the artery, potential backflow, and reflux of the embolic agent (Fig. 3C). During embolisation, the operator should perform selective angiograms, the quality of which enables not only assessment of occlusion progress but also the potential filling of collaterals. The tip of a microcatheter is placed into the IMA distal to the origin of the middle meningeal and accessory meningeal artery due to the anastomoses with the ICA (Fig. 2A) In addition to the medial and lateral branches of the sphenopalatine artery, it is also desirable to embolise the descending palatine artery (Fig. 3A). This artery originates distal to the infraorbital artery (Fig. 3A). Reflux of embolic particles into the infraorbital artery is generally considered to be safe. The penetration of microparticles into the middle deep temporal artery may cause pain and trismus after embolisation. The embolic microparticles are mixed with nondiluted contrast medium to achieve the best visibility during injection using a 1-ml syringe. The mixture should not be too concentrated and must be continually mixed because the PVA microparticles have a tendency to sediment and occlude the microcatheter. Spherical microparticles make a more stable mixture. Spherical microparticles can penetrate deeper into arteries than PVA microparticles of the same size. Particles should lodge distal enough to control haemorrhage and yet proximal enough to preserve the distal supply of the terminal alar artery, which supplies the skin of the nose. The PVA microparticles 200–500 μm in size are usually selected. It is useful to talk to the patient if he or she is awake to show any ischaemic neurological deficit as soon as possible. In the sleeping patient, we must rely on anatomical angiographic studies and a thorough knowledge of the anatomy [26].

Embolisation is halted when the peripheral branches of the sphenopalatine segment are not opacified and the arterial tree seems to be debranched (Fig. 3C). The amount of microparticles required varies. Special care must be taken as soon as the flow begins to slow because even small additional amounts of microparticles may result in unexpected arterial stasis or even reflux.

The strategy for embolisation in patients with idiopathic epistaxis relies on the identification of the side of bleeding and the collateral pathways involving the contralateral IMA and the ipsilateral facial artery (Fig. 2C). The distribution of embolised arteries varies significantly in published series. The bilateral IMAs together with the ipsilateral facial artery were embolised in 27–48 %, the bilateral IMAs in 15–35 %, and the unilateral IMA in 13–70 % of cases. The bilateral IMAs are embolised if the side of haemorrhage is unknown or bilateral IMAs and ipsilateral facial artery if the side of haemorrhage is known. There also exist published case reports describing embolisation of the ascending pharyngeal and accessory meningeal supply to the nasal fossa for the management of epistaxis [27–29].

#### Nasal Packing Removal

Two different strategies regarding nasal packing removal have been reported in the literature. Some operators remove the packs while the catheters are still in the arteries, check the nasal pathway for bleeding, and finish the procedure if the nasal pathway remains dry for 15 min. Persistent bleeding influences the decision to embolise the contralateral IMA or the ipsilateral facial artery [30]. Other practitioners leave the packs in place to facilitate the synergistic effects of external compression and devascularisation. Nasal packing removal is influenced by the duration of its placement, the severity and intractability of the epistaxis, and occasionally by the time of day the procedure is performed. Care must be taken to prevent the aspiration of pooled blood into the respiratory tract.

#### Clinical Success

The primary success rates of epistaxis embolization therapy range from 71 to 95 % in the literature (Table 4). The success rate is influenced by the embolisation protocol used. Embolisation of the bilateral distal IMAs and ipsilateral distal facial artery with 200-μm PVA microparticles significantly decreases short-term failure rates and carries a very low risk for ischemic complications. The rate of early recurrence decreases with embolisation of more arteries [22]. Patients taking oral anticoagulants or antiplatelet medication are noted to have greater rate of early rebleeding than patients not taking these medications [22].

**Table 4:** Review of studies on posterior epistaxis treated with embolisation

First author (reference)	Year^a^	No. of patients	Mean age (year)	No. of procedures	Embolic material for ECA branches	Immediate clinical success (%)^b^	Severe complications (%)^c^
Vítek [30]	1991	30	62	34	GS	87	3.3 hemiparesis
Elden [32]	1994	97	53	108	PVA	88	2 stroke, skin slough
Elahi [57]	1995	57	53.1	54	PVA	91	6 stroke
Tseng [7]	1998	112	55	114	PVA, GS	91	0.9 stroke
Moreau [58]	1998	45	48.8	46	PVA	95	4
Leppanen [59]	1999	37	53	38	PVA, coils	89	0
Oguni [60]	2000	37	57.3	40	GS	95	0
Andersen [33]	2005	22	59	30	PVA, spheres	87	5 nose necrosis
Christensen [61]	2005	70	59.1	70	PVA, GS, coils	87	1 stroke
Sadri [34]	2006	14	57	15	PVA	71	7 nose necrosis, palate necrosis
Fukutsuji [62]	2008	22	56.8	23	GS, coils	77.3	0
Strach [35]	2011	48	58.7	53	PVA, coils	93.5	4 nose necrosis, hemiparesis
Gottumukkala [56]	2013	84	63.8	85	PVA	89	1

*GS* = gelatin sponge

^a^The table includes studies with selective embolisation using microcatheters

^b^The overall clinical success also depended on the proportions of patients with HHT

^c^Definitions of severe stroke were variable

Failures of embolisation therapy to control epistaxis are due to dominant ICA supply by way of the anterior and/or posterior ethmoid arteries. This arterial supply requires external surgical ligation. Late rebleeds are dependent on the underlying pathology and are more frequent with HHT. Often these patients are included in studies involving idiopathic epistaxis [30].

#### Surgical Versus Endovascular Therapy

The decision between surgery and embolisation should be based on the individual patient’s comorbidities, the anatomical setting, and the availability of adequate interventional radiology [11]. There are retrospective studies available comparing IMA ligation (with or without ethmoid artery ligation) [11] with PVA embolisation of the bilateral IMAs and ipsilateral facial artery [31] for the treatment of refractory epistaxis. The embolised arteries were not specified in one of those studies [11]. The success rates for IMA ligation and embolisation were 89 and 94 %, respectively [31]. The other study reported success rates of 73 and 79 %, respectively [11]. The investigators in both studies concluded that complication and failure rates of IMA ligation and embolisation were comparable. However, although the major complication rates were not significantly different between the two therapies, the complications associated with embolisation were more serious than those associated with IMA ligation.

The advantages of PVA embolisation include the use of local anaesthesia; pretreatment angiogram, which may show less common causes of epistaxis; distal embolisation; and shorter hospitalisation times. Embolisation is indicated in patients with cardiovascular instability. However, embolisation is available only in specialised centres. Furthermore, if bleeding originates from the ethmoid arteries, embolisation by way of the ophthalmic artery is considered to be hazardous. Ophthalmic artery embolisation should be avoided, even in cases of long-standing blindness in the ipsilateral eye, because retinal necrosis may lead to autoimmune retinal inflammation and loss of vision in the contralateral eye [15]. In the presence of dangerous collaterals, the embolisation material must be exchanged for microcoils, which results in a more proximal and less effective embolisation or referral for surgical treatment. Surgical ligation of the IMA with or without anterior ethmoid artery ligation seems to be the more common technique and requires general anaesthesia in all cases.

### Complications

Embolisation should decrease flow to the bleeding nasal mucosa while avoiding necrosis of the nasal skin or palate mucosa. Skin necrosis requires plastic surgical repair [32–35]. Smaller particles (45–150 μm) carry a greater risk of collateral tissue ischemia, including ischemia of the vasa nervorum [36].

The most frequent causes of neurologic deficit and blindness [37] after embolisation in the ECA territory are reflux of emboli due to vasospasm, nonselective injection, injection of excessive particles at an excessive rate, and failure to recognise dangerous anastomoses due to incomplete angiograms and inadequate analysis [27, 38].

Minor complications include postembolisation ischemic syndrome, which typically lasts for a short time and requires only symptomatic therapy. Acute ischemic sialadenitis has been reported after reflux of microparticles into the proximal branches of the facial artery [39].

## Management of Angiographically Proven Causes of Epistaxis

### Traumatic Epistaxis

The clinical presentation of traumatic epistaxis is life-threatening haemorrhage at the time of trauma [40]. CT imaging is indicated in all cases of suspected arterial epistaxis. Angiogram may show focal active extravasation, pseudoaneurysm [41], or arterial pseudo-occlusion. The embolisation technique for these cases differs from that for idiopathic epistaxis. The tip of the microcatheter must be as close as possible to the arterial pathology. In the case of possibly dangerous collaterals, microcoils are preferred. The most common sources of bleeding are the sphenopalatine and facial arteries. In a significant number of cases, bilateral extravasation is present [42].

When trauma or infection [43] of the ICA arterial wall results in pseudoaneurysm formation, the patient can present with delayed epistaxis. This pseudoaneurysm should be expected in the cervical, petrous, or cavernous portions of the ICA [44]. Arterial injuries during craniofacial surgery are uncommon but have been well described [45–48].

### Juvenile Nasopharyngeal Angiofibroma and Other Tumours

Juvenile nasopharyngeal angiofibroma (JNA) is a benign hypervascular tumour that occurs in young males between 8 and 23 years of age. Symptoms are related to the size and direction of spread. These symptoms may include recurrent epistaxis (observed in 59 % of cases), which may be life-threatening. The lesion originates from the posterolateral wall of the nasal cavity in close proximity to the superior aspect of the middle sphenopalatine foramen, the attachment site of the posterior part of the middle turbinate.

The systematic angiographic protocol for JNA begins with injection in the contralateral CCA followed by injections in the ICA ipsilateral common and internal carotid arteries. Next, ipsilateral external carotid angiography for evaluation of the maxillary and ascending pharyngeal arteries is performed. The maxillary, accessory meningeal, ascending pharyngeal, and ascending palatine arteries are most commonly embolised vessels. If the tumour crosses the midline, additional embolisation of the appropriate contralateral feeders is performed.

In cases with extensive tumour spread, the supply from the ICA branches may be embolised if possible, especially if the ICA must be sacrificed before surgery. Neurologic deficit is the most feared complication of preoperative embolisation. Blindness, in particular, is of major concern as a result of involvement of the ethmoidal blood supply [38].

Since the introduction of embolisation of JNA by Roberson [49], several studies have been published proving the benefit of preoperative embolisation by decreasing blood loss and operative times. Other sinonasal neoplasms that can cause life-threatening epistaxis include nasopharyngeal carcinoma and hemangiopericytoma [13].

### Epistaxis in HHT

HHT, a hereditary disease, is associated with skin and mucosal telangiectasias and arteriovenous shunts most commonly involving the lung, brain, and liver. It is easily recognised in individuals displaying the classic triad of epistaxis, telangiectasia, and a relevant family history [50].

Epistaxis is caused by spontaneous bleeding from telangiectasias of the nasal mucosa (Fig. 2B). Recurrent epistaxis begins by the age of 10–21 years and the severity of the episodes increases with age [51].

Among numerous local and surgical treatments, transcatheter embolotherapy has been used with the intent of decreasing the number and severity of epistaxis episodes. Microparticle embolisation should decrease flow to the nasal mucosa, but care must be taken to leave the proximal arteries patent. Embolisation with coils should be avoided because the artery cannot be made again accessible distal to the proximal occlusion in recurrent epistaxis. Preventive embolisation is considered to have a questionable long-term effect [32, 52].

## Conclusion

Ninety five per cent of all epistaxis episodes have their origin in the anterior septum and are easily controlled with local endonasal therapy using anterior packing if bleeding does not resolve spontaneously. Posterior epistaxis represents 5 % of cases and is more difficult to treat. The placement of posterior nasal packs may predispose patients toward vasovagal reaction, infection, and significant discomfort [53–56]. Endoscopic ligation or electrocauterisation of the sphenopalatine and anterior ethmoidal arteries is a routine technique for local control of active bleeding. Embolisation of the distal branches of the bilateral IMAs and ipsilateral distal branches of the facial artery is the preferred therapy in patients with poor cardiovascular status because this procedure can be performed with the patient under local anaesthesia in a majority of cases [22]. Embolisation is also used in the treatment of epistaxis refractory to previous endonasal or surgical therapy, including IMA ligation. Rare but major complications of embolisation therapy are mainly due to the penetration of embolic material into the intracranial and ophthalmic arteries. Embolisation therapy is relatively contraindicated when the bleeding originates mainly from the ethmoid branches of the ophthalmic artery or in the presence of anastomoses between the ECA and the ICA. In our experience, close collaboration between the otorhinolaryngologist and the interventional radiologist provides the best chance of controlling posterior epistaxis.
